# Psychological impact of COVID-19 pandemic: A cross-sectional study of hospitalized COVID-19 patients in an urban setting, Bangladesh

**DOI:** 10.1016/j.heliyon.2022.e09110

**Published:** 2022-03-12

**Authors:** Shah Golam Nabi, Md. Utba Rashid, Soumik Kha Sagar, Prakash Ghosh, Md. Shahin, Fahdia Afroz, Irfan Nowroze Noor, Irin Hossain, Dinesh Mondal, Helal Uddin Ahmed

**Affiliations:** a Directorate General of Health Services (DGHS), Mohakhali, Dhaka 1212, Bangladesh; b Nutrition and Clinical Services Division (NCSD), International Centre for Diarrhoeal Disease Research, Bangladesh (icddr,b), Mohakhali, Dhaka 1212, Bangladesh; c National Institute of Preventive and Social Medicine (NIPSOM), Mohakhali, Dhaka 1212, Bangladesh; d National Institute of Cardiovascular Diseases, Sher-e-Bangla Nagar, Dhaka 1207, Bangladesh; e Department of Child Adolescent and Family Psychiatry, National Institute of Mental Health, Sher-e-Bangla Nagar, Dhaka 1207, Bangladesh

**Keywords:** Psychological impact, COVID-19 pandemic, Cross-sectional, Hospitalized patients, Bangladesh

## Abstract

**Aim:**

The deleterious impact of the COVID-19 pandemic on mental health has been reported by earlier studies globally. However, such studies are limited in Bangladesh; therefore, we performed a cross-sectional study to explore the psychological effects of COVID-19 among hospitalized patients.

**Methodology:**

The cross-sectional study was performed from 1^st^ June to 31^st^ October, 2020, and included a total of 503 real time RT-PCR confirmed stable hospitalized adult (aged ≥18 years) COVID-19 patients using the convenience sampling approach. However, patients with prior mental illness, unstable vital signs, severely ill, oxygen saturation <92%, impaired consciousness were excluded from the study. We collected data by using a semi-structured questionnaire including Patient Health Questionnaire-9 (PHQ-9), General Anxiety Disorder-7 (GAD-7), Insomnia Severity Index (ISI-7), and Perceived Stress Scale (PSS-10). Descriptive analysis and multivariable logistic regression were carried out to determine the mental health outcomes.

**Results:**

The study found that about 42.5 %, 30.7%, 46.7%, and 28.5% of patients suffered from moderate to severe depression, anxiety, stress, and insomnia. The physical symptoms, fever, fatigue, loss of taste or smell, blurred vision, chest pain, and diarrhoea were significantly associated with augmented mental distress among the hospitalized patients. Furthermore, depression, anxiety, stress and insomnia were strongly linked with patients’ education, occupation, infected family members, exposure to COVID-19 patients, smoking, comorbidities, infection among the neighbors or acquaintances, and preexisting stress.

**Conclusion:**

The negative psychological impact of the COVID-19 pandemic comprising depression, anxiety, insomnia and stress worsened the physical condition of hospitalized COVID-19 patients. These patients' poor mental health status needed to be addressed by devising an integrated approach towards improving patients' wellbeing at the post-COVID period.

## 1 Introduction

A cluster of mysterious pneumonia cases emerged in Wuhan city, Hubei province, China, in late 2019, caused by a virus named SARS-CoV-2. Since then, it spread worldwide abruptly, reaching the level of the pandemic as such that the World Health Organization (WHO) declared this as a “Public Health Emergency of International Concern”, thereafter being officially coined as Coronavirus disease 2019 (COVID-19) (Adhikari et al., 2020; Hawryluck et al., 2004; Qiu et al., 2020). To date, a wealth of research has been performed to understand the disease trajectories including epidemiology, pathogenesis and transmission dynamics, where endeavors are still ongoing to devise therapeutic and preventive interventions (Chen et al., 2020; Huang et al., 2020; Lu et al., 2020; Rubin and Wessely, 2020). Despite significant efforts, the scourge of the disease is continuously jeopardizing the lives and livelihoods of millions all over the world with a persistent rise in death tolls. This outbreak has a catastrophic impact on the economy as well as the terror of this pandemic has had a detrimental effect on mental health like other infectious disease outbreaks (Hall et al., 2008; Ho et al., 2020). The menace of this pandemic is putting a significant psychological impact on individual life where the magnitude of the sufferings has been reported higher among the COVID-19 infected patients (Deng et al., 2021). These aggravated sufferings among the COVID-19 patients can be attributed to the incessant mental distress owing to the uncertainty of the disease outcome, fear of death and the health complications caused by the viral infection as well. According to the previous literature, the continuous mental distress arising from the fear of death combined with severe anxiety, depression create unfavorable mental health conditions of patients, which is aggravated manifold due to insomnia and physical effects of the disease (Xiang et al., 2020; Zandifar et al., 2020a, 2020b).

Due to the pandemic, the healthcare centers have been flooded with COVID-19 patients, therefore the healthcare providers cannot offer time-efficient care where the logistics and workforce are severely limited (Arya et al., 2020). As a result, the psychological symptoms are being under-evaluated and under-managed in COVID-19 patients. Moreover, most medical practitioners prioritize physical conditions while mental health is crucial in intensifying patients’ disease outcomes, as evidenced by previous research. The previous study explored that depressed and anxious inpatients were more likely to have poorer outcomes, longer hospital stays, and a higher risk of rehospitalization compared to their counterparts (Dennis et al., 2012).

While much clinical attention and research focus has been given in treating the physiological fallout caused by the novel coronavirus, the psychological effects of the virus on infected patients are often overlooked. Recent case reports and observational studies have suggested that COVID-19 patients are likely to develop depression, anxiety, and sleep disturbances (Fu and Zhang, 2020). Therefore, to improve the disease outcome, reduce the length of hospital stay, and minimize long-term mental health difficulties, practitioners should properly evaluate and treat these conditions. Despite the importance of treating COVID-19 individuals with poor mental health status, existing research on the mental health impact of COVID-19 among the infected individuals in Bangladesh is inadequate and bleary. Eventually, tailoring an effective intervention model is imperative to mitigate the mental and psychological burden owing to this pandemic situation among the hospitalized COVID-19 infected patients. However, adequate information on psychological impact among the COVID-19 patients is a prerequisite to improvising such an intervention model considering the country's socio-economic and demographic contexts. Therefore, we undertook a cross-sectional study towards assessing the mental and psychological status of the affected COVID-19 patients.

## 2 Methodology

### 2.1 Study design, site, population and sample size

The study was a cross-sectional survey performed in four tertiary COVID-19 dedicated specialized hospitals in Dhaka. The hospitalized adult patients aged ≥18 years, stable, confirmed COVID-19 with real time reverse transcription polymerase chain reaction (real time RT-PCR) along with mild to moderate clinical symptoms (as per national guideline), free from any mental illness and willing to participate in this research were under interview. However, patients with prior mental illness, unstable vital signs, severely ill, oxygen saturation <92%, impaired consciousness, unwilling to give informed consent were excluded from the study.

According to the previous study conducted in Wuhan, we found that approximately 53.8% of the affected patients were suffering from moderate to severe psychological distress during the COVID-19 outbreak (Wang et al., 2020a). However, to conduct this study we assumed that around 53% our study participants would be suffering from mild to severe level of psychological distress such as depression, anxiety or stress. Furthermore, with a 95% level of significance and 80% error, our estimated sample size for this study was 382.

### 2.2 Study procedure and sampling

Two researchers used the convenience sampling method to complete the data collection procedure from June 01 to October 31, 2020, through a face-to-face interview. Without hampering the patient's rest, researchers approached every stable patient admitted into the hospital. They explained the purpose of the study and requested them to participate. Upon agreement, the researchers collected their socio-demographic as well as their mental health status using a semi-structured questionnaire. Two researchers cross-checked all data and a third researcher resolved any difference in interpretation between the two primary reviewers.

### 2.3 Measures

#### 2.3.1 Socio-demographics measures

Socio-demographic data were collected, including age, sex, education, occupation, number of family members and number of under-five children. In addition, data on the patient's clinical profile, alternation of daily activities, and the presence of COVID-19 stressors were also collected.

### 2.4 Patient Health Questionnaire (PHQ-9)

Depression was measured using the PHQ-9 based on the diagnostic criteria for depression from the Diagnostic and Statistical Manual of Mental Disorders, 4th Edition (DSM-IV). We used the Bangla version of PHQ-9 to evaluate the status of depression among hospitalized COVID-19 patients with high sensitivity (89.5%) and reasonable specificity (78.8%) for detecting depressive symptoms among adolescents as validated in the Bangladeshi population (Chowdhury et al., 2004; Kroenke et al., 2001; Richardson et al., 2010). This scale consists of 9 items answered on a four-point Likert scale ranging from 0 (“Not at all”) to 3 (“Nearly every day”). The total score ranged from 0 to 27, categorized into four groups ranging from minimal (0–4) to severe (20–27) based on their score. In our current study, we took the median cut-off value for depression as ≥10 (Islam et al., 2020; Kroenke et al., 2001). People suffering from moderate to severe depression were prone to develop long-term mental health complications; that is why we used the above cut-off value as ‘Depression present’. The Cronbach's alpha for this study was high (0.85).

### 2.5 Generalized anxiety disorder (GAD-7)

Likewise, the seven-item scale of GAD-7 showed reasonable specificity and sensitivity in detecting anxiety among the general population (Mossman et al., 2017). We adopted the Bangla version of this questionnaire to assess the level of anxiety of our study participants (Haque et al., 2018). This scale consists of 7 questions responded on a four-point Likert scale ranging from 0 (“Not at all”) to 3 (“Nearly every day”). The total score ranged from 0 to 21. A total score of 0–4 indicates minimal anxiety, 5–9 indicates mild anxiety, 10–14 indicates moderate anxiety and 15–21 indicates severe anxiety. For this particular scale, Cronbach's alpha was 0.84. Those who scored ≥10 in this study were graded as having anxiety positive (Islam et al., 2020; Spitzer et al., 2006).

### 2.6 Insomnia severity index (ISI-7)

Insomnia was evaluated using the Bangla Insomnia Severity Index, covering seven issues (Bhat et al., 2011). We recorded Insomnia-related problems based on the past two weeks (e.g., “How noticeable to others do you think your sleep problem is in terms of impairing the quality of your life?”, “How worried/distressed are you about your current sleep problem?”). For scorings of the items according to their perceived severity, we used a 5-point Likert-like scale. The entire score was summed from the seven things extending from 0 to 28. We used the cut-offs suggested by Bastien et al. in their research article (Bastien et al., 2001). The Cronbach's alpha was 0.88 in the present study.

### 2.7 Perceived stress scale (PSS-10)

To quantify the prevailing stress among the hospitalized COVID-19 infected patients, we utilized the validated Bangla version of 10 items perceived stress scale developed by Cohen et al. (Cohen et al., 1983; Mozumder, 2017). The PSS-10 is a self-report instrument consisting of 10 items purported to assess ‘‘how uncertain, unmanageable, and encumbered participants find their lives”. Each of the items on the PSS-10 is rated on a 5-point Likert scale, ranging from 0 (“Never”) to 4 (“Very often”). The scale comprised both positive (Q1, Q2, Q3, Q6, Q9 and Q10) and negative factors (Q4, Q5, Q7 and Q8) where the negative items' score was reversed and re-coded during analysis. Total scores ranged from 0 to 40, where 0–13, 14–26, and 27–40 are categorized as low, moderate, and high stress. The cut-offs used for this survey was ≥14 (Bhat et al., 2011). The Cronbach's alpha was 0.71.

### 2.8 Statistical analysis

Statistical analysis was performed using SPSS V26 (IBM SPSS Statistics, New York, United States). Demographic characteristics were described as median (interquartile range, IQR) for the continuous variables and frequency (percentage) for the categorical variables. The scores of the PHQ-9, GAD-7, ISI and PSS scales were expressed as mean and standard deviation. To perform the analysis, we estimated our cut off value for the depression, anxiety, insomnia and stress subscale were ≥10, ≥10, ≥15 and ≥14 respectively as evidence of depression, anxiety, insomnia and stress. We performed simple and multivariable logistic regression to calculate the odds ratios (ORs) and the corresponding 95% confidence intervals (95% CIs) to analyze the associations between socio-demographic characteristics, changes of the social activities due to COVID-19, COVID-19 related social stressors and mental health outcomes (depression, anxiety, stress and insomnia). All tests were two-tailed, with a significance level of p < 0.05.

### 2.9 Ethical considerations

All the procedures were conducted following the ethical guidelines of the Institutional Review Board (IRB)/Ethical Review Committee (ERC) of Mugda Medical College and Hospital, Bangladesh (MUMC/2020/604). The ethical standards laid down in the 1964 Declaration of Helsinki and its later amendments or comparable ethical standards were followed wherever applicable.

## 3 Result

A total of 503 COVID-19 positive patients were interviewed for this study and their socio-demographic characteristics were presented in Table 1. This study was male predominant (57.5%) with a median age of 43 years (IQR: 35–55). Almost half of the participants completed graduation and beyond (45%) where only 8% was found illiterate. Majority of the interviewee were married (82%) with a median of 5 (4–6) family members along with an under five children in the family. Most of the respondents of this study were service holders (36%) and housewives (26%). About 56% of the study population reported to have comorbidities.

**Table 1:** Characteristics of the study participants.

Variables		n (%)
Age (year), median (IQR)		43 (35–55)
Sex	Male	289 (57.5%)
	Female	214 (42.5%)
Level of education	Illiterate	39 (8%)
	Primary education	74 (15%)
	Secondary school certificate or equivalent	74 (15%)
	Higher secondary school certificate or equivalent	91 (18%)
	Graduation	149 (30%)
	Post- Graduation	76 (15%)
Marital status	Unmarried	59 (12%)
	Married	414 (82%)
	Separated/Widowed/Other	30 (6%)
Occupation	Student	31 (6%)
	Job holder	184 (36%)
	Business	79 (16%)
	Housewife	132 (26%)
	In retirement	54 (11%)
	Others (politicians, social workers etc.)	23 (5%)
Average number of family member, median (IQR)		5 (4–6)
Average number of under five children in the family, median (IQR)		1 (0–2)
Number of comorbidities	None	222 (44%)
	At least one	144 (29%)
	Two or more	137 (27%)
Personal habit (smoking)	Yes	73 (14.5%)
	No	430 (85.5%)

In Table 2, alterations of regular activities during COVID-19 were portrayed. Almost all the participants (94%) were living with family during that time. 52% reported that, their job responsibility was same as before and 33% had to go their workplace even during lockdown period. This might be a reason why 58% reported to leave their domicile at least once a day where 167 were wage earners. The ratio between social media users and non-users during this pandemic situation was found 1.4:1.

**Table 2:** Alternation of the daily activities during COVID-19.

Variables		n (%)
Living during COVID-19 period	With family	474 (94%)
	Alone	29 (6%)
Any changes in job responsibilities during COVID-19	Not applicable	102 (20)
	Same as before	260 (52)
	Work from home	79 (16)
	Need to give more time	53 (10)
	Suspended due to COVID-19	09 (2)
Have to go to the workplace during lockdown period	Not applicable	102 (20)
	Yes	167 (33)
	No	234 (47)
Number of times for leaving domicile each day	0	210 (42)
	1	200 (40)
	2+	93 (18)
Reasons for going out during lockdown period	Wage earner	167 (55)
	Fulfill daily necessities	103 (34)
	Others (treatment, bill pay)	36 (11)
Use of social media during COVID situation	Same as before	153 (30%)
	Using a bit more than before	104 (21%)
	Using much more than before	41 (8%)
	Do not use any social media	205 (41%)
Average minutes on social media (in min), Mean ± SD		44.34 ± 57.55

**Figure 1:**
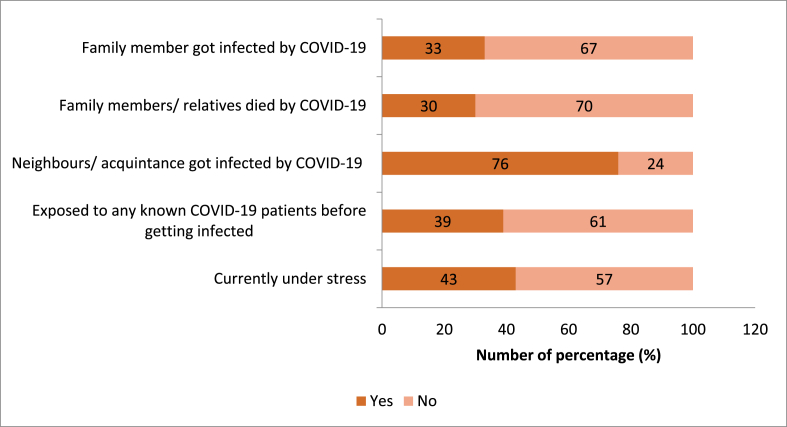
Prevalence of COVID-19 related social stressors among the infected patients.

In this study, we tried to measure the COVID-19 related stressors that usually triggered poor mental health outcomes among our study participants. The percentages of family member and neighbors of getting infected by COVID-19 was 33% and 76% respectively whereas around one-third of our participants lost their family members due to COVID-19. About 43% respondents complained of being under stress during the interview period (Figure 1).

**Figure 2:**
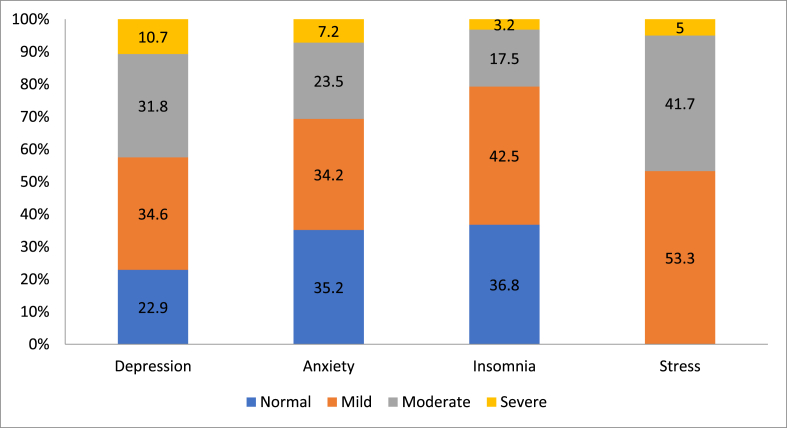
Presence of mental health outcome among the hospitalized patients.

Among all the study participants, we found that almost half of them were suffering from moderate to severe level stress (46.7%) followed by depression (42.5%), anxiety (30.7%) and insomnia (20.7%) (Figure 2).

The associations among COVID-19 symptoms among participants and mental health outcomes were represented in Table 3. Among the symptoms, blurring of vision was associated with higher level of anxiety (2.59; 1.32–5.08), insomnia (3.70; 1.86–7.36) and stress (2.91; 1.40–6.02). An interesting finding is that loss of taste/smell was associated with lower level of depression (0.54; 0.36–0.81) but higher level of stress (1.57; 1.08–2.3). Association among other symptoms and mental health outcomes include fever with stress (1.59; 1.10–2.31); fatigue with stress (1.57; 1.10–2.25); chest pain with insomnia (1.64; 1.02–2.64); diarrhoea with insomnia (1.98; 1.18–3.32); decreased mobility with anxiety (2.74; 1.82–4.12) and others (non-specific) symptoms with insomnia (2.76; 1.29–5.93).

**Table 3:** Perceived COVID-19 symptoms by the hospitalized patients.

Variables		n (%)	Depression OR (95% CI)	Anxiety OR (95% CI)	Insomnia OR (95% CI)	Stress OR (95% CI)
Fever	Yes	325 (65%)	0.72 (0.50–1.04)	0.80 (0.54–1.19)	1.54 (0.96–2.48)	1.59∗ (1.10–2.31)
	No	178 (35%)	Ref.	Ref.	Ref.	Ref.
Cough	Yes	299 (59%)	0.93 (0.65–1.33)	0.84 (0.57–1.23)	0.75 (0.49–1.16)	0.92 (0.64–1.31)
	No	204 (41%)	Ref.	Ref.	Ref.	Ref.
Sore throat	Yes	174 (35%)	1.38 (0.95–1.99)	1.03 (0.69–1.53)	0.53∗ (0.32–0.87)	0.80 (0.55–1.16)
	No	329 (65%)	Ref.	Ref.	Ref.	Ref.
Fatigue	Yes	202 (40%)	0.85 (0.59–1.21)	0.73 (0.50–1.09)	0.96 (0.62–1.50)	1.57∗ (1.10–2.25)
	No	301 (60%)	Ref.	Ref.	Ref.	Ref.
Respiratory distress	Yes	225 (45%)	1.08 (0.76–1.54)	1.26 (0.86–1.84)	1.08 (0.70–1.66)	1.29 (0.91–1.84)
	No	278 (55%)	Ref.	Ref.	Ref.	Ref.
Loss of taste/smell	Yes	154 (31%)	0.54∗∗ (0.36–0.81)	0.79 (0.52–1.21)	0.75 (0.46–1.22)	1.57∗ (1.08–2.30)
	No	349 (69%)	Ref.	Ref.	Ref.	Ref.
Muscle pain	Yes	162 (32%)	0.83 (0.57–1.22)	0.98 (0.65–1.46)	0.97 (0.61–1.55)	0.87 (0.60–1.27)
	No	341 (68%)	Ref.	Ref.	Ref.	Ref.
Chest pain	Yes	121 (24%)	0.82 (0.54–1.24)	1.28 (0.83–1.98)	1.64∗ (1.02–2.64)	1.27 (0.84–1.91)
	No	382 (76%)	Ref.	Ref.	Ref.	Ref.
Diarrhoea	Yes	87 (17%)	1.00 (0.63–1.60)	1.32 (0.81–2.15)	1.98∗ (1.18–3.32)	1.51 (0.95–2.40)
	No	416 (83%)	Ref.	Ref.	Ref.	Ref.
Headache	Yes	116 (23%)	0.68 (0.44–1.04)	0.48∗∗ (0.29–0.80)	1.14 (0.69–1.89)	0.79 (0.52–1.20)
	No	387 (77%)	Ref.	Ref.	Ref.	Ref.
Blurring of vision	Yes	37 (7%)	1.03 (0.53–2.03)	2.59∗∗ (1.32–5.08)	3.70∗∗∗ (1.86–7.36)	2.91∗ (1.40–6.02)
	No	466 (93%)	Ref.	Ref.	Ref.	Ref.
Decreased mobility	Yes	141 (28%)	1.22 (0.83–1.81)	2.74∗∗∗ (1.82–4.12)	1.40 (0.88–2.23)	1.38 (0.93–2.04)
	No	362 (72%)	Ref.	Ref.	Ref.	Ref.
Others (nausea, vomiting, back pain etc.)	Yes	30 (6%)	0.56 (0.25–1.25)	1.14 (0.52–2.50)	2.76∗∗ (1.29–5.93)	1.33 (0.63–2.78)
	No	473 (94%)	Ref.	Ref.	Ref.	Ref.
No symptoms	Yes	85 (17%)	1.32 (0.83–2.11)	1.00 (0.60–1.66)	0.79 (0.43–1.45)	0.60∗ (0.37–0.98)
	No	418 (83%)	Ref.	Ref.	Ref.	Ref.
Average time of seeking doctor's help after getting infected		3.55 ± 2.0	1.02 (0.93–1.11)	1.04 (0.95–1.14)	0.85∗∗ (0.75–0.95)	1.06 (0.97–1.15)

∗ p-value<0.05, ∗∗ p-value<0.01, ∗∗∗ p-value<0.001.

OR = Odds ratio, CI = Confidence interval.

We performed bivariate logistic regression to find out the associated factors which might be responsible for influencing mental health status in this very pandemic situation (Table 4). Depression, anxiety and insomnia were proportionately increased with the increase of the age among the hospitalized COVID-19 infected patients. The prevalence of depression, anxiety, insomnia and stress were significantly lower among the higher educated individuals than the illiterate. The presence of depression were almost 2 and 5 times higher among the married (1.85, 1.02–3.35) and widowed (4.94, 1.92–12.72) patients than the unmarried. The increased number of the family member was directly associated with the higher level of anxiety (1.13, 1.02–1.24) and stress (1.12, 1.07–1.30). In our study, presence of COVID positive family members in the family were found associated in increasing insomnia (2.21, 1.42–3.43) among the infected patients, whereas the news of affected neighbors was linked in increasing depression (1.57, 1.03–2.40) and anxiety (2.12, 1.29–3.47). We observed those who had more than one comorbidity were suffered more depression (1.84, 1.19–2.83), anxiety (2.14, 1.35–3.89) and insomnia (4.82, 2.81–8.28) than the non-comorbid patients. Interestingly, we found lower level of mental health impact among the patients who had to leave their domicile at least once in a day to fulfill their daily needs.

**Table 4:** Association between the socio-demographic status and the COVID-19 mental health impact among the study participants in bivariate logistic regression analysis.

Variable		Depression	Anxiety	Insomnia	Stress
		OR (95% CI)	OR (95% CI)	OR (95% CI)	OR (95% CI)
Age		1.03∗∗∗ (1.01–1.04)	1.02∗∗ (1.01–1.04)	1.03∗ (1.01–1.04)	1.01 (0.99–1.02)
Sex	Female	Ref.	Ref.	Ref.	Ref.
	Male	0.74 (0.52–1.06)	0.95 (0.64–1.39)	0.68 (0.44–1.05)	0.97 (0.68–1.38)
Education	Illiterate	Ref.	Ref.	Ref.	Ref.
	Primary to higher secondary	0.64 (0.32–1.26)	0.54 (0.27–1.07)	0.36 (0.18–1.74)	0.62 (0.31–1.24)
	Graduation or more	0.46∗ (0.23–0.92)	0.33∗∗ (0.17–0.67)	0.31∗∗ (0.15–0.64)	0.43∗ (0.22–0.87)
Marital Status	Unmarried	Ref.	Ref.	Ref.	Ref.
	Married	1.85∗ (1.02–3.35)	0.87 (0.48–1.56)	1.70 (0.78–3.71)	0.87 (0.50–1.50)
	Separated/Widowed	4.94∗∗ (1.92–12.72)	1.84 (0.75–4.54)	2.73 (0.93–8.04)	1.35 (0.56–3.28)
Occupation	Student	Ref.	Ref.	Ref.	Ref.
	Job holder	1.29 (0.57–2.90)	0.77 (0.32–1.86)	3.41 (0.78–14.95)	0.43∗ (0.20–0.94)
	Business	1.09 (0.45–2.64)	2.53∗ (1.01–6.34)	3.12 (0.67–14.64)	0.70 (0.30–1.63)
	Housewife	2.10 (0.92–4.80)	1.12 (0.46–2.73)	4.64∗ (1.05–20.53)	0.72 (0.33–1.59)
	In retirement	2.26 (0.90–5.69)	1.56 (0.59–4.16)	6.11∗ (1.30–28.69)	0.50 (0.20–1.22)
	Others (politicians, social workers etc.)	2.73 (0.89–8.34)	4.47∗ (1.40–14.28)	4.03 (0.71–22.99)	7.58∗ (1.51–38.18)
Average number of family member		1.09 (0.99–1.20)	1.13∗ (1.02–1.24)	1.04 (0.94–1.16)	1.12∗∗ (1.07–1.30)
Average number of under five children in the family		1.19 (0.99–1.44)	1.09 (0.90–1.33)	0.95 (0.75–1.20)	1.33∗∗ (1.09–1.61)
Family member got infected by COVID-19	No	Ref.	Ref.	Ref.	Ref.
	Yes	1.05 (0.73–1.54)	0.90 (0.60–1.35)	2.21∗∗∗ (1.42–3.43)	0.82 (0.57–1.19)
Exposed to any known COVID-19 patients before getting infected	No	Ref.	Ref.	Ref.	Ref.
	Yes	0.78 (0.54–1.12)	0.77 (0.52–1.14)	1.00 (0.65–1.56)	0.57∗ (0.34–0.82)
Comorbidities	None	Ref.	Ref.	Ref.	Ref.
	At least one	1.53 (0.99–2.35)	1.50 (0.94–2.39)	1.82∗ (1.01–3.28)	1.10 (0.72–1.68)
	Two or more	1.84∗∗ (1.19–2.83)	2.14∗∗ (1.35–3.89)	4.82∗∗∗ (2.81–8.28)	1.32 (0.86–2.03)
Personal habit (smoking)	No	Ref.	Ref.	Ref.	Ref.
	Yes	0.62 (0.37–1.05)	0.32∗ (0.16–0.63)	0.25∗ (0.10–0.63)	0.82 (0.50–1.35)
Living during COVID-19 period	Alone	Ref.	Ref.	Ref.	Ref.
	With family	3.00∗ (1.20–7.50)	2.89 (0.99–8.46)	7.77∗ (1.05–57.82)	1.47 (0.68–3.17)
Neighbors/acquaintance got infected by COVID-19	No	Ref.	Ref.	Ref.	Ref.
	Yes	1.57∗ (1.03–2.40)	2.12∗ (1.29–3.47)	1.44 (0.84–2.47)	0.88 (0.58–1.32)
Family members/relatives died by COVID-19	No	Ref.	Ref.	Ref.	Ref.
	Yes	0.95 (0.64–1.40)	1.16 (0.77–1.75)	0.90 (0.56–1.45)	0.84 (0.57–1.23)
Currently under stress	No	Ref.	Ref.	Ref.	Ref.
	Yes	1.31 (0.92–1.88)	2.53∗∗∗ (1.71–3.72)	2.08∗ (1.34–3.22)	3.12∗∗∗ (2.16–4.50)
Use of social media during COVID situation	Same as before	Ref.	Ref.	Ref.	Ref.
	Using a bit more than before	0.80 (0.48–1.34)	0.86 (0.49–1.54)	1.11 (0.59–2.11)	0.75 (0.44–1.25)
	Using much more than before	0.97 (0.48–1.96)	1.58 (0.76–3.27)	1.71 (0.76–3.83)	1.88 (0.94–3.76)
	Do not use any social media	1.47 (0.96–2.24)	1.51 (0.96–2.39)	1.35 (0.80–2.29)	1.88∗∗ (1.23–2.87)
Number of times you leave domicile each day	None	Ref.	Ref.	Ref.	Ref.
	One or more than one times/day	0.55∗∗ (0.39–0.79)	0.55∗∗ (0.37–0.81)	0.49∗∗ (0.32–0.76)	0.57∗∗ (0.40–0.82)

∗ p-value<0.05, ∗∗ p-value<0.01, ∗∗∗ p-value<0.001.

OR = Odds ratio, CI = Confidence interval.

After adjusting the variables (Table 5), the prevalence of anxiety and insomnia was significantly lower among the higher educated individuals than the illiterate. The presence of anxiety was around 7.5 times higher among the business personnel (7.68, 1.79–32.97) and the social workers (7.45, 1.62–34.36) than the student. On the other hand, homemakers and retired persons had 76% and 81% less stress than the students. In our study, the presence of COVID positive family members in the family was found associated with increasing insomnia (2.75, 1.53–4.93) among the infected patients, whereas the news of affected neighbors was linked to increasing depression (1.72, 1.05–2.81) and anxiety (2.80, 1.51–5.20). We found those who had two or more comorbidities were suffered from insomnia (3.46, 169–7.07) than the non-comorbid patients. We found that smoking reduced anxiety among the smoker (0.25, 0.10–0.60) than the non-smoker. The presence of stressful conditions due to pre-existing causes significantly deteriorated the mental health status among the affected respondents. Lastly, we observed a lower mental health impact among the patients who had to leave their domicile at least once a day to fulfill their daily needs.

**Table 5:** Association between the variables and COVID-19 mental health impact among the study participants in multivariable logistic regression analysis.

Variable		Depression	Anxiety	Insomnia	Stress
		AOR (95% CI)	AOR (95% CI)	AOR (95% CI)	AOR (95% CI)
Age		1.02 (0.992–1.04)	1.01 (0.98–1.04)	1.01 (0.98–1.04)	0.99 (0.96–1.01)
Sex	Female	Ref.	Ref.	Ref.	Ref.
	Male	1.04 (0.58–1.86)	0.53 (0.27–1.05)	0.90 (0.44–1.85)	1.09 (0.58–2.05)
Education	Illiterate	Ref.	Ref.	Ref.	Ref.
	Primary to higher secondary	1.17 (0.53–2.56)	0.42∗ (0.18–0.98)	0.29∗ (0.11–0.73)	0.94 (0.41–2.16)
	Graduation or more	0.86 (0.36–2.07)	0.25∗∗ (0.09–0.66)	0.18∗ (0.06–0.51)	0.94 (0.37–2.41)
Marital Status	Unmarried	Ref.	Ref.	Ref.	Ref.
	Married	1.57 (0.60–4.10)	0.84 (0.27–2.57)	1.14 (0.29–4.42)	2.01 (0.71–5.69)
	Separated/Widowed	2.23 (0.57–8.72)	1.20 (0.26–5.49)	1.20 (0.21–6.94)	2.88 (0.67–12.34)
Occupation	Student	Ref.	Ref.	Ref.	Ref.
	Job holder	1.20 (0.43–3.32)	1.64 (0.43–6.27)	5.47 (0.88–34.00)	0.44 (0.16–1.27)
	Business	0.76 (0.25–2.31)	7.68∗∗ (1.79–32.97)	4.32 (0.63–29.82)	0.55 (0.17–1.72)
	Housewife	0.85 (0.28–2.62)	0.32 (0.08–1.31)	1.56 (0.23–10.66)	0.24 (0.07–0.79)
	In retirement	0.93 (0.28–3.13)	0.96 (0.21–4.40)	3.19 (0.43–23.61)	0.21 (0.06–0.74)
	Others (politicians, social workers etc.)	2.36 (0.67–8.33)	7.45∗ (1.62–34.36)	3.42 (0.48–24.47)	5.35 (0.91–31.31)
Average number of family member		1.00 (0.87–1.15)	1.07 (0.91–1.26)	0.94 (0.78–1.14)	1.14 (0.99–1.32)
Average number of under five children in the family		1.10 (0.84–1.46)	0.84 (0.61–1.17)	0.82 (0.57–1.18)	1.10 (0.82–1.47)
Family member got infected by COVID-19	No	Ref.	Ref.	Ref.	Ref.
	Yes	0.93 (0.60–1.47)	0.80 (0.47–1.38)	2.75∗∗ (1.53–4.93)	0.76 (0.47–1.24)
Exposed to any known COVID-19 patients before getting infected	No	Ref.	Ref.	Ref.	Ref.
	Yes	0.78 (0.51–1.19)	0.77 (0.47–1.27)	0.91 (0.52–1.59)	0.57∗ (0.36–0.90)
Comorbidities	None	Ref.	Ref.	Ref.	Ref.
	At least one	1.11 (0.68–1.81)	1.24 (0.68–2.25)	1.27 (0.64–2.51)	1.05 (0.63–1.77)
	Two or more	1.09 (0.62–1.90)	1.34 (0.70–2.60)	3.46∗∗ (1.69–7.07)	1.20 (0.66–2.18)
Personal habit (smoking)	No	Ref.	Ref.	Ref.	Ref.
	Yes	0.99 (0.53–1.85)	0.25∗ (0.10–0.60)	0.36 (0.12–1.03)	0.82 (0.43–1.59)
Living during COVID-19 period	Alone	Ref.	Ref.	Ref.	Ref.
	With family	1.68 (0.58–4.91)	0.91 (0.24–3.39)	1.89 (0.21–16.75)	0.90 (0.31–2.60)
Neighbors/acquaintance got infected by COVID-19	No	Ref.	Ref.	Ref.	Ref.
	Yes	1.72∗ (1.05–2.81)	2.80∗∗ (1.51–5.20)	1.33 (0.69–2.57)	1.22 (0.73–2.03)
Family members/relatives died by COVID-19	No	Ref.	Ref.	Ref.	Ref.
	Yes	0.72 (0.46–1.13)	0.93 (0.56–1.57)	0.65 (0.36–1.17)	0.85 (0.53–1.37)
Currently under stress	No	Ref.	Ref.	Ref.	Ref.
	Yes	1.22 (0.83–1.80)	2.99∗∗∗ (1.89–4.75)	2.86∗∗∗ (1.70–4.80)	3.05∗∗∗ (2.03–4.58)
Use of social media during COVID situation	Same as before	Ref.	Ref.	Ref.	Ref.
	Using a bit more than before	0.71 (0.41–1.24)	0.81 (0.42–1.56)	1.08 (0.52–2.23)	0.62 (0.34–1.10)
	Using much more than before	0.99 (0.46–2.13)	1.80 (0.74–4.36)	2.25 (0.83–6.10)	1.72 (0.76–3.88)
	Do not use any social media	0.85 (0.49–1.49)	0.87 (0.45–1.70)	0.67 (0.32–1.40)	1.80 (0.99–3.25)
Number of times you leave domicile each day	None	Ref.	Ref.	Ref.	Ref.
	One or more than one times/day	0.54∗ (0.32–0.92)	0.33∗∗ (0.18–0.62)	0.50∗ (0.26–0.95)	0.43∗ (0.24–0.77)

∗ p-value<0.05 , ∗∗ p-value<0.01, ∗∗∗ p-value<0.001.

AOR = Adjusted odds ratio, CI = Confidence interval.

## 4 Discussion

This is one of the very first attempts to assess the mental health conditions (depression, anxiety and stress) including insomnia among hospital admitted COVID-19 patients in Bangladesh. We found that about 43% of the participants had moderate to severe depressive symptoms, which is comparable to that of China (45%) (Deng et al., 2021), Iran (38%) (Zarghami et al., 2020) and Italy (38%) (Liguori et al., 2020). Albeit an adjacent country, the prevalence of depression among the COVID-19 patients in India was found two times lower (Kumar et al., 2021). Study findings also indicate that 31% of the respondents had anxiety symptoms into variable severities. The earlier studies performed in Iran (29%) and Italy (33%) reported an almost similar level of anxiety that the COVID-19 patients have experienced enrolled in the current study. However, the anxiety observed among the patients was markedly higher among the Chinese (48%) and Indian (63%) populations. In this study, we have found that about 21% of the hospital admitted patients had moderate to severe levels of insomnia. This rate is also different from that of China (54.1%) (Hu et al., 2020), Turkey (13%) (Karadaş et al., 2020) and Italy (50%) (Liguori et al., 2020) in the perspective of severity where some of them reported mild to severely insomniac. An essential finding of this current study is that almost half of the interviewed patients’ (47%) reported stress symptoms in variable degrees. At the time of the SARS outbreak in 2001, a study in Hong Kong revealed that hospitalized patients had an increased rate of stress and adverse psychological effects (Chua et al., 2004). It is proven that disease-related isolation is one of the major causes of psychological symptoms among patients (Xiang et al., 2020). Being a life-threatening disease, COVID-19 is supposed to cause panic and stress among patients in isolation (Lim et al., 2020).

Our study found a significant association between education level and mental health outcomes among our study population. It is found that highly educated individuals had 58–82% lower rates of mental distress than participants with no educational backgrounds. However, discrepancies have been observed in previous studies. A nationwide survey was done in Bangladesh among the general population that depicted higher rates of stress among graduate individuals (Banna et al., 2020), where few studies reported that lower education level was associated with higher stress, anxiety, insomnia and depression (Mazza et al., 2020; Olagoke et al., 2020). Higher educated people had more access to authentic news sources than illiterate participants. We assumed that a sound knowledge regarding disease transmission, disease manifestation, and disease containment could be the driving factors for lowering depression, anxiety, stress, and insomnia among the higher educated people.

Our current analysis found that anxiety was significantly higher among the business personnel and those who earned their livings daily. Our study was in line with the previous survey conducted on the Bangladeshi population (Haque et al., 2020). The garment sector is one of the core components of our national economy. This sector was severely affected due to disruptions of supply chains. National lockdowns across export destinations led to buyers canceling or postponing orders, resulting in factories shut down and unemployment (Hasan, 2020). Small businesses also faced multiple challenges (e.g., decreased market demand) due to the COVID-19 pandemic. Many business owners even failed to meet daily costs. The continuation of the pandemic forced a majority of the small businesses to close down, which was the employment of source for 50 million people (Khalil, 2020). Lack of financial certainty during the national emergency might create tremendous mental pressure on them, whereas job holders or government employees had the slightest possibility of job discontinuation (Posel et al., 2021). Due to the pandemic and national lockdown, most of the COVID-19 participants who earned daily failed to secure sufficient income to afford their families. Moreover, the scenario became more complicated after being hospitalized due to COVID-19. All of these were responsible for creating anxiety among them.

The participants exposed to the confirmed COVID-19 individual before getting affected were found 43% less likely to have stress than the participants who were not. Our result was in line with the survey performed by Wang C et al. on the Chinese population (Wang et al., 2020a). As our COVID-19 cases are escalating day by day, the burden of asymptomatic individuals is uprising. Nowadays, it is difficult to prevent the contact of asymptomatic patients roaming around us. The survey jointly conducted by icddr,b, and IEDCR reported that among COVID-19 cases, 82% of patients did not report any COVID-like symptoms (icddrb, 2020). The abundance of these asymptomatic cases and lack of proper community case detection might be the possible reason for creating more stress among unexposed study participants than exposed ones, who were aware of the possible consequences.

According to the previous findings, comorbidities significantly augment the disease severity of COVID-19 patients. Likewise, our study reported that patients with more than one comorbidity suffered higher levels of insomnia. Experience of poor health and fear of being infected might be the possible reasons predicted in previous research (Rozario and Masho, 2018). In addition, patients with chronic diseases felt more vulnerable to diseases due to poor immunity and self-evaluation of such poor health, which was also associated with adverse mental health outcomes (Ho et al., 2020; Holmes et al., 2020; Wang et al., 2020b).

Furthermore, we found that participants who used to leave their domicile for various reasons had around 45% less depression, anxiety, stress and insomnia than those confined at home. This finding was inverse to that of the study conducted by Mazza C et al., where leaving domicile contributed to stress (Mazza et al., 2020). Considering Bangladesh ought economic context, most people to go outside to earn their bread and butter. Staying at home made them more concerned about their provisions than the fear of being infected with the COVID-19.

The further analysis explored that infected individuals in the family and/or neighbors significantly augmented the level of depression, anxiety, and insomnia in COVID-19 hospitalized patients. This finding is congruent with previous studies on different populations (Abir et al., 2021; Fegert et al., 2020; Mazza et al., 2020; Wang et al., 2020a). Ours is a densely populated country where different variants of coronavirus prevail at a time. Moreover, the features of each strain are unique. Besides, the asymptomatic cases have worsened our current situation and played a critical role in community transmission. From that perspective, an increased number of infected neighbors and family members might cause tremendous psychological pressure among the affected hospitalized patients.

Interestingly, the patients who had the habit of smoking were 75% less likely to have anxiety than the non-smokers. According to the Mental Health Foundation report, UK, people currently under any anxiety or stress are likely to smoke two times higher than those without any mental anxiety (Mental Health foundation, 2021). This finding is unique because prior studies reported that bad personal habits are associated with adverse mental conditions (Simonsick, 1991). As nicotine can relax nerves, smokers probably used it as a way out to reduce their mental distress during this pandemic situation. This phenomenon needs to be evaluated in the future.

We found that patients who had a history of stressful events or went through pre-existing stressful situations before getting infected had significantly increased anxiety, stress, and insomnia levels. According to previous research, a history of trauma exerts a significant long-term sequel of mental strain and tension among the sufferers (Wiseman et al., 2015). So, pre-existing stress could be one of the underlying factors which played a critical role in aggravating the psychological symptoms while staying in the hospital.

In this study, 94% of the hospitalized patients reported having at least one symptom related to COVID-19. Logistic regression elucidated that physical symptoms (e.g., fever, fatigue, loss of smell/taste) were significantly associated with a higher level of stress, often observed as the first symptoms. These findings align with a study conducted among Bangladeshi students (Khan et al., 2020). A fascinating finding is that blurring of vision is highly associated with a higher level of anxiety, insomnia, and stress. This finding is unique as no authors had reported this relationship previously. Patients who had no symptoms were found to have a lower stress level, which is quite understandable because they felt their conditions were less severe than the symptomatic patients.

Since in this study, the mental health status of COVID-19 affected hospitalized patients have been evaluated for the first time, eventually it provides indispensable information considering the country's perspectives which would guide to establish proper intervention strategies in the future. In addition, this study has been conducted in multiple sites where we included significant numbers of patients for a face-to-face interview; therefore, data generated from this study has the generalizability for Dhaka city with broader implications.

With no exception, this study has several limitations. Firstly, this study was conducted on only clinically stable patients with COVID-19, as the severe and life-threatening condition of COVID-19 patients precluded them from participating in this survey. Secondly, this study relied more on self-reported answers instead of mental health professionals' clinical diagnoses, which might not portray the absolute mental health condition of the patients. Another limitation is that we did not have baseline pre-pandemic data of the enrolled participants; therefore, we cannot nullify the impact of individuals' preexisting mental health conditions. Moreover, the study was conducted only in the COVID-19 dedicated tertiary hospital of Dhaka city during a complete lockdown, which might fail to portray the actual mental health condition of the COVID-19 affected hospitalized patients of Bangladesh. Last but not least, it was a cross-sectional design; due to the lack of a control group, we could not establish the causal relationships among the variables. Notwithstanding the above limitations, this study provides invaluable information on COVID-19 affected hospitalized in-patients' psychological responses during the COVID-19 pandemic.

## 5 Conclusion

The results evinced that a significant proportion of COVID-19 patients experienced a varying degree of moderate to severe depression, anxiety, insomnia and perceived stress during hospitalization. The cause of these psychiatric disorders is likely to be multifactorial. Lack of contact with families and loved ones during quarantine or hospitalization, age, comorbidities, the concern of family members getting infected, lack of access to social media, and misinformation regarding COVID-19 may facilitate the development of psychiatric disorders in COVID-19 patients. The high prevalence and severity of these psychiatric disorders further emphasize the need to pay more attention to their mental health along with their physical improvement. Further demonstration of suicide and suicidal tendencies of COVID-19 patients prioritized the importance of early detection and intervention to this vulnerable cohort (Akhter, 2021; Bagcchi, 2020; bdnews24.com, 2021; Liu et al., 2020). The poor mental health status observed among the hospitalized COVID-19 patients necessitates utmost attention from healthcare providers and policymakers to ensure their wellbeing through psychological care and timely intervention. Hospitals should integrate distant psychosocial screening and post-COVID psychiatric counseling through telemedicine and online mental health sessions and develop a system to allow proper communication with these hospitalized patients and their families. Further improvement of health literacy and broadcasting the correct information may also help curb social stigma surrounding COVID-19 patients and eventually upgrade their psychological wellbeing.

## Declarations

### Author contribution statement

Shah Golam Nabi, Md. Utba Rashid: Conceived and designed the experiments; Performed the experiments; Analyzed and interpreted the data; Contributed reagents, materials, analysis tools or data; Wrote the paper.

Soumik Kha Sagar, Irfan Nowroze Noor, Irin Hossain: Conceived and designed the experiments; Performed the experiments; Contributed reagents, materials, analysis tools or data.

Prakash Ghosh: Conceived and designed the experiments; Performed the experiments; Wrote the paper.

Md. Shahin, Fahdia Afroz: Performed the experiments; Contributed reagents, materials, analysis tools or data.

Dinesh Mondal: Conceived and designed the experiments; Analyzed and interpreted the data; Wrote the paper.

Helal Uddin Ahmed: Conceived and designed the experiments; Analyzed and interpreted the data; Contributed reagents, materials, analysis tools or data; Wrote the paper.

### Funding statement

This research did not receive any specific grant from funding agencies in the public, commercial, or not-for-profit sectors.

### Data availability statement

Data will be made available on request.

### Declaration of interests statement

The authors declare no conflict of interest.

### Additional information

No additional information is available for this paper.

## References

[bib1] Abir T., Kalimullah N.A., Osuagwu U.L., Nur-A Yazdani D.M., Husain T., Goson P.C., Basak P., Rahman M.A., Al Mamun A., Permarupan P.Y., Khan M.Y.H., Milton A.H., Agho K.E. (2021). Prevalence and factors associated with mental health impact of COVID-19 pandemic in Bangladesh: a survey-based cross-sectional study. Ann. Glob. Health.

[bib2] Adhikari S.P., Meng S., Wu Y.-J., Mao Y.-P., Ye R.-X., Wang Q.-Z., Sun C., Sylvia S., Rozelle S., Raat H., Zhou H. (2020). Epidemiology, causes, clinical manifestation and diagnosis, prevention and control of coronavirus disease (COVID-19) during the early outbreak period: a scoping review. Infect. Dis. Poverty.

[bib3] Akhter S. (2021).

[bib4] Arya A., Buchman S., Gagnon B., Downar J. (2020). Pandemic palliative care: beyond ventilators and saving lives. Can. Med. Assoc. J..

[bib5] Bagcchi S. (2020). Stigma during the COVID-19 pandemic. Lancet Infect. Dis..

[bib6] Banna M.H. Al, Sayeed A., Kundu S., Christopher E., Hasan M.T., Begum M.R., Kormoker T., Dola S.T.I., Hassan M.M., Chowdhury S., Khan M.S.I. (2020). The impact of the COVID-19 pandemic on the mental health of the adult population in Bangladesh: a nationwide cross-sectional study. Int. J. Environ. Health Res..

[bib7] Bastien C.H., Vallières A., Morin C.M. (2001). Validation of the Insomnia Severity Index as an outcome measure for insomnia research. Sleep Med..

[bib8] bdnews24.com (2021).

[bib9] Bhat R.M., Sameer M.K., Ganaraja B. (2011). Eustress in education: analysis of the perceived stress score (PSS) and blood pressure (BP) during examinations in medical students. J. Clin. Diagn. Res..

[bib10] Chen N., Zhou M., Dong X., Qu J., Gong F., Han Y., Qiu Y., Wang J., Liu Y., Wei Y., Xia J., Yu T., Zhang X., Zhang L. (2020). Epidemiological and clinical characteristics of 99 cases of 2019 novel coronavirus pneumonia in Wuhan, China: a descriptive study. Lancet.

[bib11] Chowdhury A.N., Ghosh S., Sanyal D. (2004). Bengali adaptation of brief patient health questionnaire for screening depression at primary care. J. Indian Med. Assoc..

[bib12] Chua S.E., Cheung V., McAlonan G.M., Cheung C., Wong J.W.S., Cheung E.P.T., Chan M.T.Y., Wong T.K.W., Choy K.M., Chu C.M., Lee P.W.H., Tsang K.W.T. (2004). Stress and psychological impact on SARS patients during the outbreak. Can. J. Psychiatr..

[bib13] Cohen S., Kamarck T., Mermelstein R. (1983). A global measure of perceived stress. J. Health Soc. Behav..

[bib14] Deng J., Zhou F., Hou W., Silver Z., Wong C.Y., Chang O., Huang E., Zuo Q.K. (2021). The prevalence of depression, anxiety, and sleep disturbances in COVID-19 patients: a meta-analysis. Ann. N. Y. Acad. Sci..

[bib15] Dennis M., Kadri A., Coffey J. (2012). Depression in older people in the general hospital: a systematic review of screening instruments. Age Ageing.

[bib16] Fegert J.M., Vitiello B., Plener P.L., Clemens V. (2020). Challenges and burden of the Coronavirus 2019 (COVID-19) pandemic for child and adolescent mental health: a narrative review to highlight clinical and research needs in the acute phase and the long return to normality. Child Adolesc. Psychiatr. Ment. Health.

[bib17] Fu R., Zhang Y. (2020). Case report of a patient with suspected COVID-19 with depression and fever in an epidemic stress environment. Gen. Psychiatr..

[bib18] Hall Ryan C.W., Hall Richard C.W., Chapman M.J. (2008). The 1995 Kikwit Ebola outbreak: lessons hospitals and physicians can apply to future viral epidemics. Gen. Hosp. Psychiatr..

[bib19] Haque M.J., Das C.K., Ara R., Alam M.E.U., Ullah S., Hossain Z.M.M. (2018). Prevalence of generalized anxiety disorder and its effect on daily living in the rural community of Rajshahi. TAJ J. Teach. Assoc..

[bib20] Haque M.N., Ansar S. Bin, Biswas G., Islam M.R., Al Mamun A. (2020). The impact of COVID-19 on socio economic condition of city people: lessons from the selected KCC area. J. Eng. Sci..

[bib21] Hasan M. (2020).

[bib22] Hawryluck L., Gold W.L., Robinson S., Pogorski S., Galea S., Styra R. (2004). SARS control and psychological effects of quarantine, Toronto, Canada. Emerg. Infect. Dis..

[bib23] Ho C.S., Chee C.Y., Ho R.C. (2020). Mental health strategies to combat the psychological impact of COVID-19 beyond paranoia and panic. Ann. Acad. Med. Singapore.

[bib24] Holmes E.A., O’Connor R.C., Perry V.H., Tracey I., Wessely S., Arseneault L., Ballard C., Christensen H., Cohen Silver R., Everall I., Ford T., John A., Kabir T., King K., Madan I., Michie S., Przybylski A.K., Shafran R., Sweeney A., Worthman C.M., Yardley L., Cowan K., Cope C., Hotopf M., Bullmore E. (2020). Multidisciplinary research priorities for the COVID-19 pandemic: a call for action for mental health science. Lancet Psychiatr..

[bib25] Hu Y., Chen Y., Zheng Y., You C., Tan J., Hu L., Zhang Z., Ding L. (2020). Factors related to mental health of inpatients with COVID-19 in Wuhan, China. Brain Behav. Immun..

[bib26] Huang C., Wang Y., Li X., Ren L., Zhao J., Hu Y., Zhang L., Fan G., Xu J., Gu X., Cheng Z., Yu T., Xia J., Wei Y., Wu W., Xie X., Yin W., Li H., Liu M., Xiao Y., Gao H., Guo L., Xie J., Wang G., Jiang R., Gao Z., Jin Q., Wang J., Cao B. (2020). Clinical features of patients infected with 2019 novel coronavirus in Wuhan, China. Lancet.

[bib27] icddrb (2020).

[bib28] Islam S., Akter R., Sikder T., Griffiths M.D. (2020). Prevalence and factors associated with depression and anxiety among first-year university students in Bangladesh: a cross-sectional study. Int. J. Ment. Health Addiction.

[bib29] Karadaş Ö., Öztürk B., Sonkaya A.R. (2020). A prospective clinical study of detailed neurological manifestations in patients with COVID-19. Neurol. Sci..

[bib30] Khalil T. (2020).

[bib31] Khan A.H., Sultana M.S., Hossain S., Hasan M.T., Ahmed H.U., Sikder M.T. (2020). The impact of COVID-19 pandemic on mental health & wellbeing among home-quarantined Bangladeshi students: a cross-sectional pilot study. J. Affect. Disord..

[bib32] Kroenke K., Spitzer R.L., Williams J.B.W. (2001). The PHQ-9. J. Gen. Intern. Med..

[bib33] Kumar P., Chaudhary R., Chhabra S., Bhalla J. (2021). Prevalence of anxiety and depression among COVID-19 patients admitted to tertiary care hospital. Indian J. Soc. Psychiatr..

[bib34] Liguori C., Pierantozzi M., Spanetta M., Sarmati L., Cesta N., Iannetta M., Ora J., Mina G.G., Puxeddu E., Balbi O., Pezzuto G., Magrini A., Rogliani P., Andreoni M., Mercuri N.B. (2020). Subjective neurological symptoms frequently occur in patients with SARS-CoV2 infection. Brain Behav. Immun..

[bib35] Lim J., Jeon S., Shin H.Y., Kim M.J., Seong Y.M., Lee W.J., Choe K.W., Kang Y.M., Lee B., Park S.J. (2020). Case of the index patient who caused tertiary transmission of coronavirus disease 2019 in Korea: the application of lopinavir/ritonavir for the treatment of COVID-19 pneumonia monitored by quantitative RT-PCR. J. Kor. Med. Sci..

[bib36] Liu Y., Cao L., Li X., Jia Y., Xia H. (2020). Awareness of mental health problems in patients with coronavirus disease 19 (COVID-19): a lesson from an adult man attempting suicide. Asian J. Psychiatr..

[bib37] Lu R., Zhao X., Li J., Niu P., Yang B., Wu H., Wang W., Song H., Huang B., Zhu N., Bi Y., Ma X., Zhan F., Wang L., Hu T., Zhou H., Hu Z., Zhou W., Zhao L., Chen J., Meng Y., Wang J., Lin Y., Yuan J., Xie Z., Ma J., Liu W.J., Wang D., Xu W., Holmes E.C., Gao G.F., Wu G., Chen W., Shi W., Tan W. (2020). Genomic characterisation and epidemiology of 2019 novel coronavirus: implications for virus origins and receptor binding. Lancet.

[bib38] Mazza C., Ricci E., Biondi S., Colasanti M., Ferracuti S., Napoli C., Roma P. (2020). A nationwide survey of psychological distress among Italian people during the covid-19 pandemic: immediate psychological responses and associated factors. Int. J. Environ. Res. Publ. Health.

[bib39] Mental Health foundation U. (2021). https://www.mentalhealth.org.uk/a-to-z/s/smoking-and-mental-health.

[bib40] Mossman S.A., Luft M.J., Schroeder H.K., Varney S.T., Fleck D.E., Barzman D.H., Gilman R., DelBello M.P., Strawn J.R. (2017). The Generalized Anxiety Disorder 7-item scale in adolescents with generalized anxiety disorder: signal detection and validation. Ann. Clin. Psychiatr..

[bib41] Mozumder M.K. (2017). Validation of Bengali perceived stress scale among LGBT population. BMC Psychiatr..

[bib42] Olagoke A.A., Olagoke O.O., Hughes A.M. (2020). Exposure to coronavirus news on mainstream media: the role of risk perceptions and depression. Br. J. Health Psychol..

[bib43] Posel D., Oyenubi A., Kollamparambil U. (2021). Job loss and mental health during the COVID-19 lockdown: evidence from South Africa. PLoS One.

[bib44] Qiu J., Shen B., Zhao M., Wang Z., Xie B., Xu Y. (2020). A nationwide survey of psychological distress among Chinese people in the COVID-19 epidemic: implications and policy recommendations. Gen. Psychiatr..

[bib45] Richardson L.P., McCauley E., Grossman D.C., McCarty C.A., Richards J., Russo J.E., Rockhill C., Katon W. (2010). Evaluation of the Patient Health Questionnaire-9 Item for detecting major depression among adolescents. Pediatrics.

[bib46] Rozario S.S., Masho S.W. (2018). The associations between mental health status, hypertension, and hospital inpatient visits in women in the United States. Am. J. Hypertens..

[bib47] Rubin G.J., Wessely S. (2020). The psychological effects of quarantining a city. BMJ.

[bib48] Simonsick E.M. (1991). Personal health habits and mental health in a national probability sample. Am. J. Prev. Med..

[bib49] Spitzer R.L., Kroenke K., Williams J.B.W., Löwe B. (2006). A brief measure for assessing generalized anxiety disorder: the GAD-7. Arch. Intern. Med..

[bib50] Wang C., Pan R., Wan X., Tan Y., Xu L., Ho C.S., Ho R.C. (2020). Immediate psychological responses and associated factors during the initial stage of the 2019 coronavirus disease (COVID-19) epidemic among the general population in China. Int. J. Environ. Res. Publ. Health.

[bib51] Wang C., Pan R., Wan X., Tan Y., Xu L., Ho C.S., Ho R.C. (2020). Immediate psychological responses and associated factors during the initial stage of the 2019 coronavirus disease (COVID-19) epidemic among the general population in China. Int. J. Environ. Res. Publ. Health.

[bib52] Wiseman T.A., Curtis K., Lam M., Foster K. (2015). Incidence of depression, anxiety and stress following traumatic injury: a longitudinal study. Scand. J. Trauma Resuscitation Emerg. Med..

[bib53] Xiang Y.T., Yang Y., Li W., Zhang L., Zhang Q., Cheung T., Ng C.H. (2020). Timely mental health care for the 2019 novel coronavirus outbreak is urgently needed. Lancet Psychiatr..

[bib54] Zandifar A., Badrfam R., Mohammadian Khonsari N., Assareh M., Karim H., Azimzadeh M., Noori Sepehr M., Tajbakhsh R., Rahimi F., Ghanipour N., Agoushi A., Hassani Gelsefid S., Etemadi F., Qorbani M. (2020). COVID-19 and medical staff’s mental health in educational hospitals in Alborz Province, Iran. Psychiatr. Clin. Neurosci..

[bib55] Zandifar A., Badrfam R., Mohammadian Khonsari N., Mohammadi M.R., Asayesh H., Qorbani M. (2020). Prevalence and associated factors of posttraumatic stress symptoms and stigma among health care workers in contact with COVID-19 patients. Iran. JAMA Psychiatr..

[bib56] Zarghami A., Farjam M., Fakhraei B., Hashemzadeh K., Yazdanpanah M.H. (2020). A report of the telepsychiatric evaluation of SARS-CoV-2 patients. Telemed. e-Health.

